# Z-Shaped Transfer Functions for Binary Particle Swarm Optimization Algorithm

**DOI:** 10.1155/2020/6502807

**Published:** 2020-06-08

**Authors:** Sha-sha Guo, Jie-sheng Wang, Meng-wei Guo

**Affiliations:** ^1^School of Electronic and Information Engineering, University of Science & Technology Liaoning, Anshan 114044, China; ^2^National Financial Security and System Equipment Engineering Research Center, University of Science & Technology Liaoning, Anshan 114044, China

## Abstract

Particle swarm optimization (PSO) algorithm is a swarm intelligent searching algorithm based on population that simulates the social behavior of birds, bees, or fish groups. The discrete binary particle swarm optimization (BPSO) algorithm maps the continuous search space to a binary space through a new transfer function, and the update process is designed to switch the position of the particles between 0 and 1 in the binary search space. Aiming at the existed BPSO algorithms which are easy to fall into the local optimum, a new Z-shaped probability transfer function is proposed to map the continuous search space to a binary space. By adopting nine typical benchmark functions, the proposed Z-probability transfer function and the V-shaped and S-shaped transfer functions are used to carry out the performance simulation experiments. The results show that the proposed Z-shaped probability transfer function improves the convergence speed and optimization accuracy of the BPSO algorithm.

## 1. Introduction

The particle swarm optimization (PSO) algorithm is one of the widely used evolutionary algorithms inspired by animal social behaviors [1, 2]. It has the search speed, high efficiency, simple algorithm, and so on and has been widely used in crystal structure prediction [3], medical detection [4], grid scheduling [5], robot path planning [6], clustering problem [7], neural network, and many other areas [8–10]. However, many optimization problems have binary searching space, so it is necessary to develop binary optimization algorithm to solve them.

The binary particle swarm optimization (BPSO) algorithm was proposed by Kennedy and Eberhart in 1997 [11]. Typical binary particle swarm optimization algorithm has two different components: a new transfer function and a different location update program. The transfer function is used to map the continuous search space to a binary space, and the update process is designed to switch the position of the particle between 0 and 1 in the binary searching space. The original version of BPSO algorithm is prone to fall into local extreme, so many scholars have made some improvements to BPSO algorithm. In 2008, Yin proposed a new improved BPSO algorithm, which uses the new updated position formula digital curve with the best polygon approximation [12]. In 2013, Wang et al. [13] used the average information of the individual population and the individual extremum of the particle swarm to determine the current probability value of the particle and removed the influence of the current value of the particle on the next generation. At the same time, the greedy thought is added so that the algorithm not only has the overall optimization characteristics of the particle swarm optimization algorithm but also accelerates the convergence speed of the algorithm. In 2014, Luo [14] revised the sigm function, taking the particle velocity as the correction term of the particle position, fully considering the guiding effect between the particles, and ensuring that the particle follows the optimization mode of the algorithm and applies it to solve the vehicle. On the path problem, the effectiveness of the algorithm is improved. Similarly, many other algorithms reference transfer functions, which are converted to binary versions by their successive versions. In 2013, Sharafi et al. [15] added a transfer function in the tracking mode of cat swarm algorithm, changed the meaning of velocity vector into the probability of mutation in each dimension of cats, and transformed continuous cat swarm algorithm into discrete binary cat swarm algorithm. In 2014, Mirjalili et al. [16] proposed a binary version of the bat algorithm, which is also a probability value that maps velocity values to updated locations using a transfer function. In the same year, Sabba and Chikhi [17] proposed a discrete binary bat algorithm (BINBA) for solving binary space optimization problems. The algorithm is based on the sigmoid function used by Kennedy and Eberhart in the binary particle swarm optimization algorithm proposed in 1997 [11]. BBA was tested on an example of a multidimensional knapsack problem. Compared with other bionic algorithms, the results have a good application prospect. In 2017, Fei [18] proposed a V-shaped transfer function to improve the binary bat algorithm to ensure that the probability of reducing the position of the vector of the position vector of the bat is an element equal to the current best position, and increasing the probability of changing the element of their position vector to the element of the bat is the element of the unequal current optimal position, which helps to enhance the optimization of the binary bat algorithm. In 2015, Emary et al. [19] proposed two new binary wolf optimization algorithms; one of which is to use the sigmoid function to compress the positions of consecutive updates, and then randomly these values are thresholded to obtain an updated double-valued gray wolf position. This method is used for binary gray wolf optimization (BGWO) to find feature subsets to maximize classification accuracy while minimizing the number of affected features. In 2017, Panwar et al. [20] proposed a heuristic binary method for solving the unit commitment problem (UC). This method estimates the continuous and valuable update of the wolves to the global optimal solution, followed by the sigmoid transformation. The simulation results show that BGWO has better performance in solving small- and medium-sized system UC problems compared with other existing heuristic and binary methods. In 2019, Hussien et al. [21] modified the original version of WOA to handle binary optimization problems. To this end, two transfer functions (s-shaped and v-shaped) are proposed to map a continuous search space to a search space. In order to illustrate the function and performance of the proposed binary whale optimization algorithm (BWOA) and apply it to 22 objective functions, 3 engineering optimization problems, and a worldwide sales problem, the results are obtained and the validity is verified. In the same year, Reddy et al. [22] mapped the binary natural PBUC problem to a continuous, real-valued whale position/location mapped to a binary search space through various transformation functions. They introduced three variants of BWOA, using a hyperbolic function, an inverse tangent function, and a sigmoid transfer function, respectively. The convergence characteristics, quality of the solution, and consistency of the results of different BWOA variables are discussed, and the superiority and statistical significance of the proposed method over the existing methods are given.

This shows that the transfer function is the most important part of the binary version of the algorithm. In this paper, a new Z-shaped transfer function is proposed and applied to the particle swarm optimization algorithm. The simulation results show that the new transfer function improves the convergence speed and optimization precision of the algorithm. The structure of this paper is organized as follows.  Section 2 introduces the standard particle swarm algorithm.  Section 3 introduces the binary particle swarm algorithm.  Section 4 proposes a new Z-shaped transfer function. In Section 5, the effectiveness of the improved algorithm is verified by simulation experiments on typical test functions.  Section 6 summarizes the full text and proposes future research directions.

## 2. Particle Swarm Optimization Algorithm

The particle swarm optimization (PSO) algorithm is an intelligent optimization algorithm proposed by Kennedy and Eberhart and Beheshti and Shamsuddin [1, 2] in 1995. It simulates the bird's flight foraging behavior and optimizes the swarm through collective collaboration between birds. In the PSO algorithm, the potential solution to each optimization problem is a bird in the search space, named as a particle. Later, Shi and Eberhart added a new impact factor *w*, which improved the detection and exploratory, and formed the current standard particle swarm optimization algorithm [23]. All particles have an appropriate value determined by an optimized function, and each particle has a velocity that determines the direction and distance of their flight. Then, the particle follows the current optimal particle to search in the solution space.

The particle's velocity and position are updated by

(1) $$\begin{array}{c} v_{i\;d}=w*v_{i\;d}+c_{1}r_{1}\left(p_{i\;d}-x_{i\;d}\right)+c_{2}r_{2}\left(p_{g\;d}-x_{i\;d}\right),\\ x_{i\;d}=x_{i\;d}+v_{i\;d}, \end{array}$$

where *v*_*i* *d*_ is the velocity of the *i*th particle in the *d*th dimension, *p*_*i* *d*_ is the optimal position of the *i*th particle so far, *x*_*i* *d*_ is the position of the current *i*th particle in the *d*th dimension, *p*_*g* *d*_ is the optimal position that the particle swarm has searched so far, and *w* is the inertia weight. In this paper, the linear decreasing weight is used, namely, *w*_max_ − ((*t∗*(*w*_max_ − *w*_min_))/*t*_max_), where *w*_max_ represents the maximum value of inertia weight, *w*_min_ represents the minimum value of inertia weight, *t* represents the current number of iterations, and *t*_max_ represents the maximum number of iterations. This principle is still used in binary PSO algorithm. *r*_1_ and *r*_2_ are two randomly generated acceleration weight coefficients between [0, 1], *c*_1_ and *c*_2_ are acceleration factors, and the value 2 is taken in this paper [24].

There are three parts in the velocity updating formula of particles. The first part is inertia or momentum, which reflects the movement habit of particles, and represents the tendency of particles to maintain their previous velocity. The second part is the cognitive part, which reflects the particle's memory or recall of its own historical experience, and represents that the particle tends to approach the optimal position of its own history. The third part is the social part, which reflects the collective historical experience of cooperation and knowledge sharing among particles, and represents the tendency of particles to approach the optimal historical position of the community.

## 3. Binary Particle Swarm Optimization Algorithm

In the binary particle swarm optimization (BPSO) algorithm, the velocity update equation has not changed, but a new velocity transfer function has been introduced to map the original continuous search space to the binary search space. The transfer function concept was originally proposed by Kennedy and Eberhart [11], which allows PSO algorithm to run in the binary searching space. In this version, particles can only be zero or 1 by taking their position vector. The effect of velocity is to indicate the probability that the bits take 0 or 1, so they propose a Sigmoid transfer function, as shown in equation (2), which can convert all real values of velocity into probability values [0, 1]:

(2) $$\begin{array}{c} T\left(v_{i}^{k}\left(t\right)\right)=\frac{1}{1+e^{-v_{i}^{k}\left(t\right)}}, \end{array}$$

where *v*_*i*_^*k*^(*t*) represents the velocity of particle *i* at iteration *t* in *k* dimension.

After converting the velocity to a probability value, the position vector can be updated with the probability of its velocity by

(3) $$\begin{array}{c} x_{i}^{k}\left(t+1\right)=\left\{\begin{array}{cc} 0, & \text{If rand}<T\left(v_{i}^{k}\left(t\right)\right),\\ 1, & \text{If rand}\geq T\left(v_{i}^{k}\left(t\right)\right), \end{array}\right. \end{array}$$

where *v*_*i*_^*k*^(*t*) represents the velocity of particle *i* at iteration *t* in *k* dimension.

**Figure 1:**
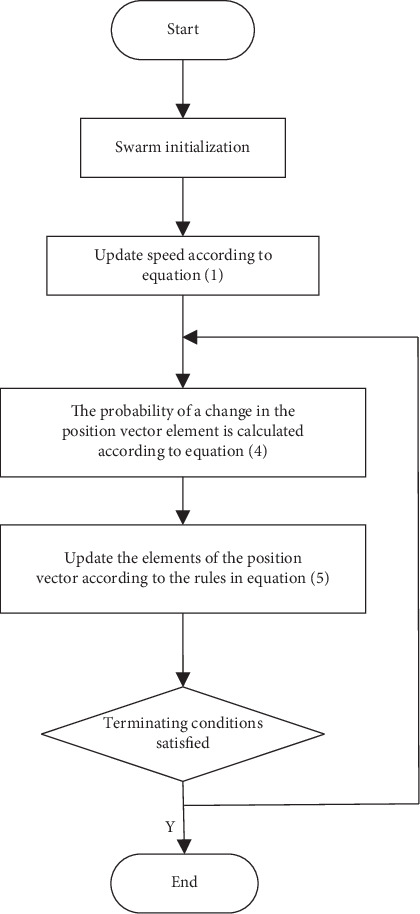
Flow chart of BPSO algorithm.

The flowchart of BPSO algorithm is shown in Figure 1. According to the experimental analysis, the original BPSO has some shortcomings, such as premature convergence and easy to fall into local optimal, so it has been continuously improved since BPSO was proposed. In 2008, Lee et al. proposed another modification of BPSO algorithm [25], which allows the continuous update velocity and position of PSO algorithm. In this improvement, they replace the velocity with the displacement in the transfer function. The probability formula is described as follows:

(4) $$\begin{array}{c} T\left(x^{k}\left(t\right)\right)=\frac{1}{1+e^{-x_{i}^{k}\left(t\right)}}, \end{array}$$

where *x*_*i*_^*k*^(*t*) represents the position of particle *i* at iteration *t* in *k* dimension.

The corresponding position updating formula is defined as follows:

(5) $$\begin{array}{c} x_{i}^{k}\left(t+1\right)=\left\{\begin{array}{cc} 0, & \text{If rand}<T\left(x_{i}^{k}\left(t\right)\right),\\ 1, & \text{If rand}\geq T\left(x_{i}^{k}\left(t\right)\right), \end{array}\right. \end{array}$$

where *x*_*i*_^*k*^(*t*) represents the position of particle *i* at iteration *t* in *k* dimension.

**Figure 2:**
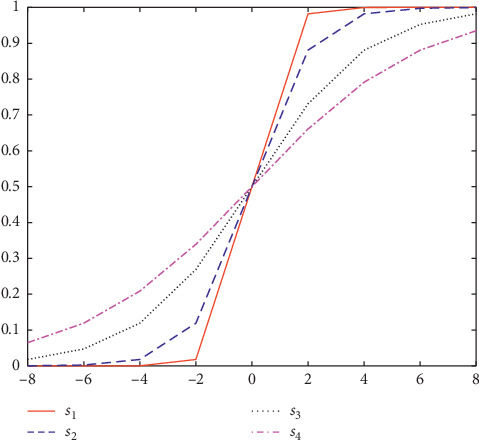
S-shaped transfer functions.

**Figure 3:**
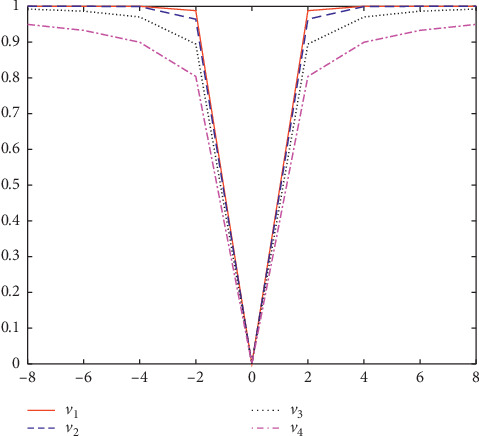
V-shaped transfer functions.

This transfer function is named as S-shaped transfer function, and a set of S-shaped transfer functions are formed by changing parameters, whose expressions and graphs are shown in Table 1 and Figure 2, respectively [24]. It can be seen from Figure 3 that the velocity value of *s*_1_ is larger than that of *s*_2_, and the saturation speed is also accelerated. Similarly, when the speed becomes smaller, as shown in *s*_3_ and *s*_4_, the saturation speed will decrease. To sum up, the change probability of position vector increases with the increase of slope of these functions. Therefore, when the velocity is the same, the probability value returned by *s*_1_ is the largest. In the following Section 5, the influence of different slopes on solving the function optimization problem will be studied.

In 2009, Rashedi et al. proposed a new transfer function named as V-shaped transfer function and a new position update strategy [26]. The formula is described as follows:

(6) $$\begin{array}{c} x_{i}^{k}\left(t+1\right)=\left\{\begin{array}{cc} {\left(x_{i}^{k}\left(t+1\right)\right)}^{-1}, & \text{If rand}<T\left(x_{i}^{k}\left(t\right)\right),\\ x_{i}^{k}\left(t\right), & \text{If rand}\geq T\left(x_{i}^{k}\left(t\right)\right), \end{array}\right. \end{array}$$

where *x*_*i*_^*k*^(*t*) represents the velocity of particle *i* at iteration *t* in *k* dimension and (*x*_*i*_^*k*^(*t*+1))^−1^ is the complement of *x*_*i*_^*k*^(*t*).

According to the characteristics of V-shaped transfer function, a series of V-shaped transfer functions are proposed by using different functional equations, whose expressions and graphs are shown in Table 2 and Figure 3, respectively.

According to Figure 3, this function is a symmetric function. When the absolute value of velocity is larger, the probability of particle position change is higher. For different V-shaped functions, it is easy to find that when the slope of the function is higher, the probability of the particle position change is higher. In other words, when the velocity is constant, the function with higher slope has a higher probability of the returned particle change. Similarly, the influence of different slopes on the processing of function optimization problems will be analyzed in Section 5.

## 4. Improved Binary Particle Swarm Optimization Algorithm

**Figure 4:**
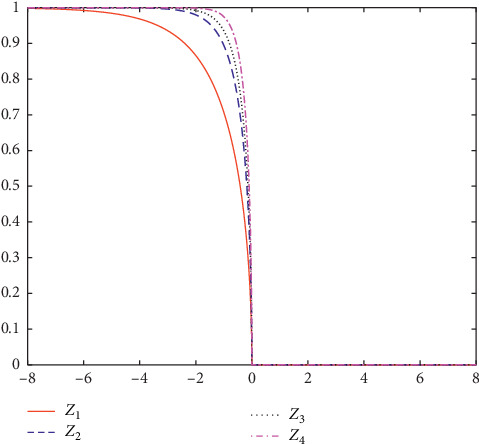
Z-shaped transfer functions.

Based on the characteristics of binary particle swarm optimization algorithm, the continuous search space is mapped to the discrete binary space. The purpose of the transfer function is to represent the probability that the element of the position vector goes from 0 to 1, so the transfer function must be a bounded function of [0, 1]. In addition, when the velocity value is 0, the probability of change should be relatively small because when the particle finds the optimal value, the velocity should be reduced to 0, and the probability of the position change of the particle should be 0. According to the characteristics of transfer function, a new Z-shaped transfer function is proposed, which is defined as follows:

(7) $$\begin{array}{c} T\left(x_{i}^{k}\left(t\right)\right)=\sqrt{1-a^{x_{i}^{k}\left(t\right)}}, \end{array}$$

where *x*_*i*_^*k*^(*t*) represents the velocity of particle *i* at iteration *t* in *k* dimension and *a* is a positive integer. By changing the value of *a*, a set of Z-shaped function families is obtained, whose expressions and figures are shown in Table 3 and Figure 4, respectively.

As shown in Figure 4, the mapping function is an asymmetric mapping function. The asymmetric mapping function basically satisfies the absolute value of the velocity to determine the mapping probability of the particle position vector variation, so the convergence speed is fast. When the parameter *Di* *m*_*particle*_=*Di* *m*_*Function*_ × 15 changes, the slope of the function also changes. The larger *Di* *m*_*particle*_=*Di* *m*_*Function*_ × 15, the smaller the slope of the function. That is to say, when the speed is the same, the probability that the returned particle position of the parameter *Di* *m*_*particle*_=*Di* *m*_*Function*_ × 15 is small changes is larger.

## 5. Simulation Experiments and Result Analysis

### 5.1. Selection of Test Functions

**Figure 5:**
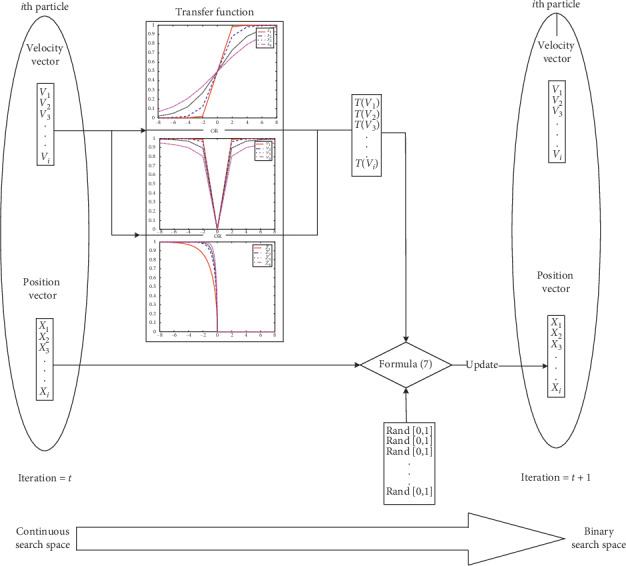
Procedure of mapping continuous search space to discrete search space.

The process of mapping continuous search space to discrete search space using the proposed transfer function is shown in Figure 5. To represent each continuous variable in binary, 15 bits are adopted. It should be noted that each function variable retains a bit of its sign. Therefore, the particle size is calculated by

(8) $$\begin{array}{c} {\text{Dim}}_{\text{practicle}}={\text{Dim}}_{\text{Function}}\times 15. \end{array}$$

**Figure 6:**
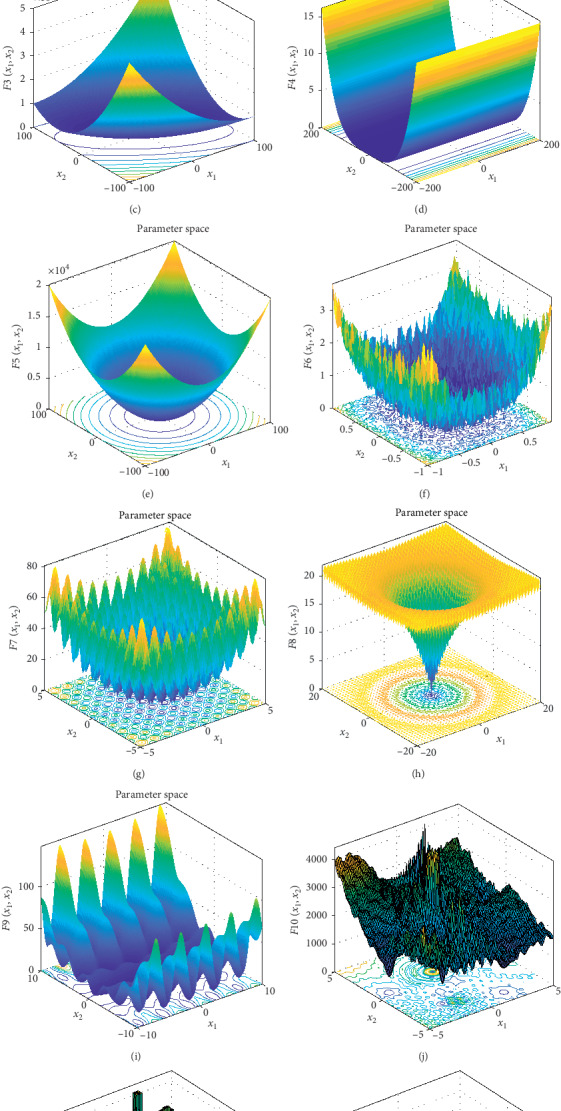
3D graphs of benchmark functions. (a) *F*_1_. (b) *F*_2_. (c) *F*_3_. (d) *F*_4_. (e) *F*_5_. (f) *F*_6_. (g) *F*_7_. (h) *F*_8_. (i) *F*_9_. (j) *F*_10_. (k) *F*_11_. (l) *F*_12_.

According to equation (8), when the test function has 5, 10, and 30 dimensions, the corresponding particle sizes are 75, 150, and 450, respectively. In order to evaluate the improved BPSO algorithm based on the Z-shaped transfer functions, the simulation experiments were carried out by using the nine benchmark functions proposed in the CEC special conference. The expressions of the benchmark functions and the three-dimensional graphics are shown in Table 4 and Figure 6, respectively.

### 5.2. Performance Comparison of Z-Shaped Transfer Functions

**Figure 7:**
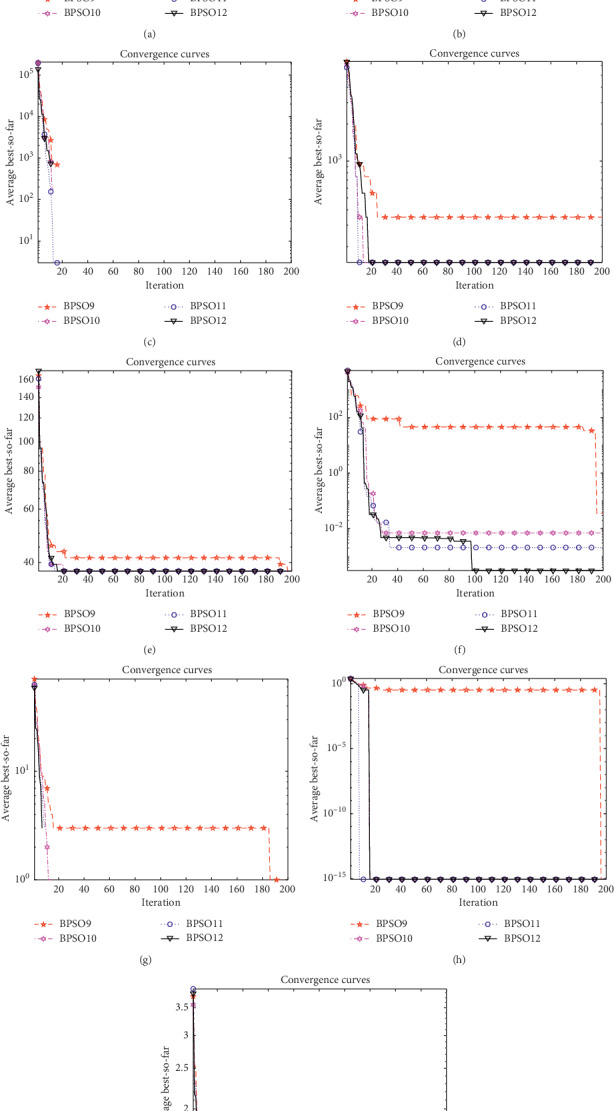
Convergence curves of the benchmark functions under *D*=5. (a) *F*_1_. (b) *F*_2_. (c) *F*_3_. (d) *F*_4_. (e) *F*_5_. (f) *F*_6_. (g) *F*_7_. (h) *F*_8_. (i) *F*_9_.

**Figure 8:**
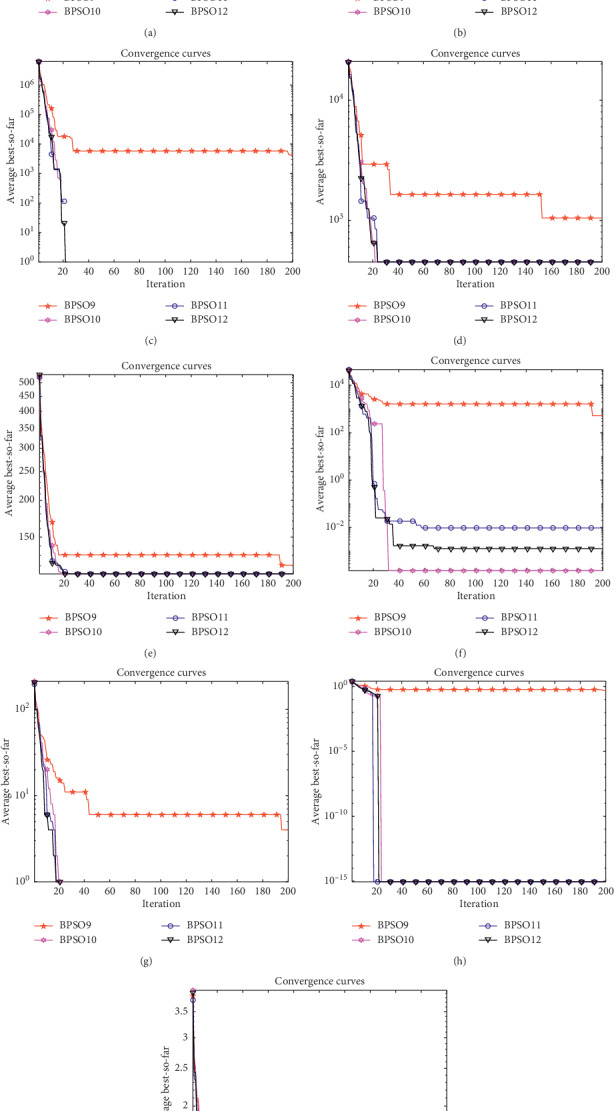
Convergence curves of the benchmark functions under *D*=30. (a) *F*_1_. (b) *F*_2_. (c) *F*_3_. (d) *F*_4_. (e) *F*_5_. (f) *F*_6_. (g) *F*_7_. (h) *F*_8_. (i) *F*_9_.

In order to test the performance of the Z-shaped transfer function, the parameters of the Z-shaped transfer function are set as 2, 5, 8, and 20, respectively, and the influence of different parameter settings on the simulation experiments is analyzed. In order to further verify the optimization accuracy of Z-shaped transfer function, each test function was run 10 times, and the best, worst, average, and STD values of 9 functions in 5 and 30 dimensions were recorded, respectively. The maximum number of iterations is Max_iter=500. The simulation results of *D*=5 and *D*=30 are shown in Figures 7 and 8, respectively, and the statistical results are shown in Tables 5 and 6, respectively.

It can be seen from Figure 7 and Table 5 that when the test functions have 5 dimensions, changing the parameters has no effect on the final convergence results, and the final convergence results are the same. However, the rate of convergence will be different. In the function convergence curves, it can be seen that increasing the value of parameters can improve the convergence speed of the algorithm, but it is not the case that the larger the parameters are, the faster the convergence speed will be. In the convergence curves of *F*_1_, *F*_2_, *F*_3_, *F*_7_, and *F*_9_, the transfer function whose parameter is set to 20 has the fastest convergence speed. In the convergence curves of *F*_4_, *F*_5_, and *F*_8_, the transfer function with parameter set to 8 has the fastest convergence speed.

It can be seen from Figure 8 and Table 6 that when the test functions have 30 dimensions, changing the size of parameters has no obvious influence on the convergence speed of the algorithm, but has an influence on the final convergence result. In the functions *F*_1_, *F*_2_, *F*_3_, *F*_4_, *F*_5_, *F*_7_, *F*_8_, and *F*_9_, the parameters are set to 5, 8, and 20, and the effect is better when the parameter is 2, that is to say that increasing the parameter can improve the accuracy of the algorithm. However, when the parameters are set to 5, 8, and 20, the results are the same. That is to say, it is not the case that the larger the parameters setting, the better the performance. When the parameters are set to a certain size, increasing the parameters will not improve the accuracy of the algorithm.

### 5.3. Performance Comparison of Z-Shaped, S-Shaped, and V-Shaped Transfer Functions

#### 5.3.1. Test Function Results and Analysis

**Figure 9:**
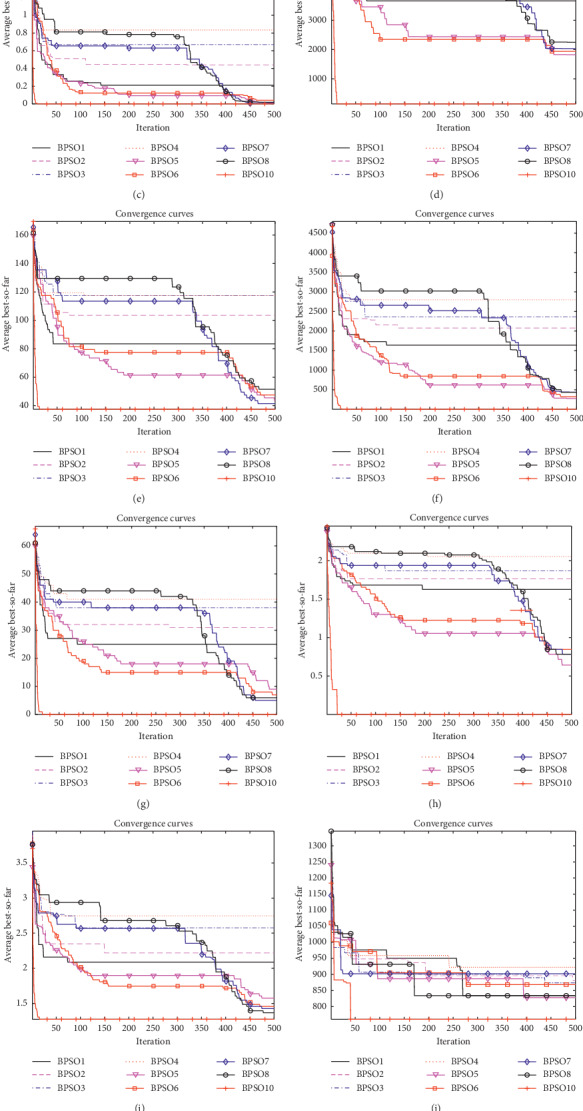
Convergence curves of the benchmark functions. (a) *F*_1_. (b) *F*_2_. (c) *F*_3_. (d) *F*_4_. (e) *F*_5_. (f) *F*_6_. (g) *F*_7_. (h) *F*_8_. (i) *F*_9_. (j) *F*_10_. (k) *F*_11_. (l) *F*_12_.

To further investigate the validity of the Z-shaped transfer function, the Z-shaped transfer function is compared to the S-shaped and the V-shaped transfer function. Through the above analysis, it can be concluded that when the parameter of the Z-shaped transfer function is set to 5, the convergence precision and convergence speed are optimal. Therefore, in this set of simulation experiments, the parameter of the new Z-shaped transfer function is set as 5, the selected 12 test functions have 10 dimensions, and the maximum number of iterations is 500. The simulation results and numerical analysis are shown in Figure 9 and Table 7, respectively.

It can be seen from the convergence curves that the proposed new transfer function improves the convergence speed of the algorithm. According to the data shown in Table 7, the new transfer function improves the accuracy of the BPSO algorithm. In particular, for functions *F*_1_, *F*_2_, *F*_3_, and *F*_7_, the optimal value of the function can be accurately derived. Similarly, for functions *F*_8_, the accuracy is 15 orders of magnitude higher than other transfer functions. For the composite functions *F*_10_ ~ *F*_12_, BPSO10 improves the convergence accuracy and convergence speed of the algorithm, but the optimal value of the function cannot be found, which will be the focus of future research and learning. In other functions, it also improves the convergence speed and convergence precision of the algorithm compared to other transfer functions.

#### 5.3.2. Nonparametric Test Analysis

In order to verify the stability of the algorithm, we perform a nonparametric test on the optimal value of the test function. In this work, Wilcoxon rank sum test [27] is used as a nonparametric statistical test to determine the importance of the results.  Table 8 depicts the 5% *p* value obtained from this test. It can be seen from Table 8 that the *p* values for all test functions are much less than 0.05, highlighting the significant advantages of BPSO10 over other versions of the binary particle swarm algorithm based on the *p* value (less than 0.05) algorithm.

### 5.4. Comparison with Other Versions of Binary Particle Swarm Algorithms

In order to further investigate the effectiveness of the transfer function, this paper compares the test results of BPSO10 with other recent binary particle swarm versions. Firstly, it was compared with the binary algorithm (BPSOGSA) proposed by Mirjalili et al. [28] based on particle swarm optimization algorithm and gravity search algorithm. The dimension of the test function was 5, and the comparison result was shown in Table 9. At the same time, it also proposed binary hybrid topology particle swarm optimization (BHTPSO) and binary hybrid topology particle swarm optimization quadratic interpolation (BHTPSO-QI), proposed by Beheshti et al. [29], and swarm optimization algorithm based on genetic algorithm is put forward by the quantum-behaved particle swarm optimization (SOGA) by Sun et al. [30] are compared. The dimension of the test function was 8 dimensions, and the comparison result is shown in Table 10.

It can be seen from the data in Tables 9 and 10 that when the test function is 5 or 8 dimensions, the test results of the newly proposed z-type transfer function are significantly higher than those of BPSOGSA, BHTPSO, BHTPSO-QI, and SOGA proposed in recent studies. In particular, in the test functions *F*_1_, *F*_2_, and *F*_3_, BPSO10 can still accurately find the optimal value, and the STD data shows that the value is 0, indicating that the algorithm is very stable and has strong robustness. Among other test functions, BPSO10 also improves the optimization accuracy of the algorithm to varying degrees. Therefore, the newly proposed z-type transfer function has good optimization ability.

## 6. Conclusions

In this paper, a new Z-shaped transfer function is proposed to improve BPSO algorithm. The simulation results on three sets' data show that the newly proposed Z-shaped transfer function improves the convergence speed and convergence precision of BPSO algorithm. In addition, by changing the slope of the Z-shaped transfer function, it is found that when the test function dimension is low, the slope has no effect on the final convergence result, but it will affect the convergence speed. When the test function dimension is high, the slope has no effect on the convergence speed. However, it will affect the convergence result. Combined with three sets of experiments, it is found that when the parameter is set as 5, the convergence speed is the fastest and the precision is highest. In the future research, BPSO algorithm can be improved by using the new transfer function as a direction of exploration. In addition, certain family functions with different slopes are used for comparative study to find the optimal algorithm.

## Figures and Tables

**Table 1:** S-shaped transfer functions.

Experiment name	Name	Expression
BPSO1	*s*_1_	*T*_1_(*x*)=(1/(1+*e*^−2*x*^))
BPSO2	*s*_2_	*T*_2_(*x*)=(1/(1+*e*^−*x*^))
BPSO3	*s*_3_	*T*_3_(*x*)=(1/(1+*e*^(−*x*/2)^))
BPSO4	*s*_4_	*T*_4_(*x*)=(1/(1+*e*^(−*x*/3)^))

**Table 2:** V-shaped transfer functions [24].

Experiment name	Name	Expression
BPSO5	*V*_1_	$T_{5}\left(x\right)=\left|\text{erf}\left(\left(\sqrt{\pi }/2\right)x\right)\right|$
BPSO6	*V*_2_	*T*_6_(*x*)=|tanh(*x*)|
BPSO7	*V*_3_	$T_{7}\left(x\right)=\left|\left(x\right)/\sqrt{1+x^{2}}\right|$
BPSO8	*V*_4_	*T*_8_(*x*)=|(2/*π*)arctan((*π*/2)*x*)|

**Table 3:** Z-shaped transfer functions.

Experiment name	Name	Expression
BPSO9	*Z*_1_	$T_{9}\left(x\right)=\sqrt{1-2^{x}}$
BPSO10	*Z*_2_	$T_{10}\left(x\right)=\sqrt{1-5^{x}}$
BPSO11	*Z*_3_	$T_{11}\left(x\right)=\sqrt{1-8^{x}}$
BPSO12	*Z*_4_	$T_{12}\left(x\right)=\sqrt{1-20^{x}}$

**Table 4:** Test functions.

Function	Dim	Rang	F_min_
*F*_1_(*x*)∑_*i*=1_^*n*^*x*_*i*_^2^	5, 8, 10, 30	[−100, 100]	0
*F*_2_(*x*)∑_*i*=1_^*n*^|*x*_*i*_|+∏_*i*=1_^*n*^|*x*_*i*_|	5, 8, 10, 30	[−10, 10]	0
*F*_3_(*x*)=∑_*i*=1_^*n*^(∑_*j*−1_^*n*^*x*_*j*_)^2^	5, 8, 10, 30	[100, 100]	0
*F*_4_(*x*)=∑_*i*=1_^*n*−1^[100(*x*_*i*+1_ − *x*_*i*_^2^)^2^+(*x*_*i*_ − 1)^2^]	5, 8, 10, 30	[−30, 30]	0
*F*_5_(*x*)=∑_*i*=1_^*n*^[(*x*_*i*_+5)]^2^	5, 8, 10, 30	[−100, 100]	0
*F*_6_(*x*)=∑_*i*=1_^*n*^(*ix*_*i*_)^4^+random[0,1)	5, 8, 10, 30	[−1.28, 1.28]	0
*F*_7_(*x*)=∑_*i*=1_^*n*^[*x*_*i*_^2^ − 10 cos(2*πx*_*i*_)+10]	5, 8, 10, 30	[−5.12,−5.12]	0
$F_{8}\left(x\right)=-20\;\exp\;\times \left(-0.2\sqrt{1/n}\sum_{i=1}^{n}x_{i}\right)-\exp\left(1/n\sum_{i=1}^{n}\cos\left(2\pi x_{i}\right)\right)+20+e$	5, 8, 10, 30	[−32, 32]	0
*F*_9_=(*π*/*n*){10 sin(*πy*_1_)+∑_*i*=1_^*n*−1^(*y*_*i*_ − 1)^2^[1+10 sin^2^(*πy*_*i*_+1)]+(*y*_*n*_ − 1)^2^}+∑_*i*=1_^*n*^*u*(*x*_*i*_, 10,100,4) $y_{i}=1+\frac{x_{i}+1}{4}$	5, 8, 10, 30	[−50, 50]	0
$u\left(x_{i},a,k,m\right)=\left\{\begin{array}{cc} k{\left(x_{i}-a\right)}^{m} & x_{i}>a\\ 0 & -a<x_{i}<a\\ k{\left(-x_{i}-a\right)}^{m} & x_{i}<-a \end{array}\right.$			
*F*_10_ [24]:	10	[−5, 5]	360
*f*_1_ ~ *f*_2_ = Rotated Expanded Scaffer's FF Function			
Where FF is Shifted Rosenbrock.			
*f*_3_ ~ *f*_4_ = Rastrigin's Function			
*f*_5_ ~ *f*_6_ = FF4 Function			
*f*_7_ ~ *f*_8_ = Weierstrass Function			
*f*_9_ ~ *f*_10_ = Griewank's Function			
*σ*=[1,1,1,1,1,2,2,2,2,2]			
*λ*=[5*∗*5/100; 5/100; 5*∗*1; 1; 5*∗*1; 1; 5*∗*10; 5*∗*5/200; 5/200]			
*F*_11_ [24]	10	[−5, 5]	360
All settings are the same as *F*_10_			
$x_{j}=\left\{\begin{array}{cc} x_{j} & \left|x_{j}-o_{1j}\right|<1/2\\ \text{round}\left(2x_{j}\right)/2 & \left|x_{j}-o_{1j}\right|\geq 1/2 \end{array}\right.$ for *j* = 1, 2,…, *n*			
Except $\text{round}\left(x\right)=\left\{\begin{array}{cc} a-1 & \text{if}\;x\leq 0\;\&\;b\geq 0.5\\ 1 & \text{if}\;b<0.5\\ a1 & x>0\;\&\;b\geq 0.5 \end{array}\right.$			
Where *a* is *x*'*s* integral part and *b* is *x*'*s* decimal part.			
*F*_12_ [24]	10	[−5, 5]	260
*f*_1_ = Weierstrass Function			
*f*_2_ = Rotated Expanded Scaffer's FF Function			
Where FF is Shifted Rosenbrock.			
*f*_3_ = FF4 Function			
Where FF is Rastrigin's Function			
*f*_4_ = Ackley's Function			
*f*_5_ = Rastrigin's Function			
*f*_6_ = Griewank's Function			
*f*_7_ = Noncontinuous Expanded Scaffer's FF Function			
Where FF is Shifted Rosenbrock.			
*f*_8_ = Noncontinuous Rastrigin's Function			
*f*_9_ = High Conditioned Elliptic Function			
*f*_10_ = Sphere Function with Noise in Fitness			
*σ*_*i*_=2, for *i* = 1, 2,…, *n*			
*λ*=[10; 5/20; 1; 5/32; 1; 5/100; 5/50; 1; 5/100; 5/100]			

**Table 5:** Numerical results under *D*=5.

Function	Arithmetic	Best	Worst	Ave	Std
*F*_1_	BPSO9	0.0000	0.0000	0.0000	0.0000
	BPSO10	0.0000	0.0000	0.0000	0.0000
	BPSO11	0.0000	0.0000	0.0000	0.0000
	BPSO12	0.0000	0.0000	0.0000	0.0000

*F*_2_	BPSO9	0.0000	0.0000	0.0000	0.0000
	BPSO10	0.0000	0.0000	0.0000	0.0000
	BPSO11	0.0000	0.0000	0.0000	0.0000
	BPSO12	0.0000	0.0000	0.0000	0.0000

*F*_3_	BPSO9	0.0000	0.0000	0.0000	0.0000
	BPSO10	0.0000	0.0000	0.0000	0.0000
	BPSO11	0.0000	0.0000	0.0000	0.0000
	BPSO12	0.0000	0.0000	0.0000	0.0000

*F*_4_	BPSO9	74.0000	74.0000	74.0000	0.0000
	BPSO10	74.0000	74.0000	74.0000	0.0000
	BPSO11	74.0000	74.0000	74.0000	0.0000
	BPSO12	74.0000	74.0000	74.0000	0.0000
*F*_5_	BPSO9	18.7500	18.7500	18.7500	0.0000
	BPSO10	18.7500	18.7500	18.7500	0.0000
	BPSO11	18.7500	18.7500	18.7500	0.0000
	BPSO12	18.7500	18.7500	18.7500	0.0000

*F*_6_	BPSO9	0.0039	0.0836	0.0279	0.0219
	BPSO10	0.0002	0.0130	0.0044	0.0043
	BPSO11	0.0001	0.0043	0.0018	0.0013
	BPSO12	0.0001	0.0024	0.0010	0.0008

*F*_7_	BPSO9	0.0000	0.0000	0.0000	0.0000
	BPSO10	0.0000	0.0000	0.0000	0.0000
	BPSO11	0.0000	0.0000	0.0000	0.0000
	BPSO12	0.0000	0.0000	0.0000	0.0000

*F*_8_	BPSO9	8.8818*E* − 16	8.8818*E* − 16	8.8818*E* − 16	0.0000
	BPSO10	8.8818*E* − 16	8.8818*E* − 16	8.8818*E* − 16	0.0000
	BPSO11	8.8818*E* − 16	8.8818*E* − 16	8.8818*E* − 16	0.0000
	BPSO12	8.8818*E* − 16	8.8818*E* − 16	8.8818*E* − 16	0.0000

*F*_9_	BPSO9	1.3744	1.3744	1.3744	0.0000
	BPSO10	1.3744	1.3744	1.3744	0.0000
	BPSO11	1.3744	1.3744	1.3744	0.0000
	BPSO12	1.3744	1.3744	1.3744	0.0000

**Table 6:** Numerical results under *D*=30.

Function	Arithmetic	Best	Worst	Ave	Std
*F*_1_	BPSO9	2.0000	7.0000	4.2000	1.4697
	BPSO10	0.0000	0.0000	**0.0000**	0.0000
	BPSO11	0.0000	0.0000	**0.0000**	0.0000
	BPSO12	0.0000	0.0000	**0.0000**	0.0000

*F*_2_	BPSO9	2.0000	8.0000	4.5000	1.9621
	BPSO10	0.0000	0.0000	**0.0000**	0.0000
	BPSO11	0.0000	0.0000	**0.0000**	0.0000
	BPSO12	0.0000	0.0000	**0.0000**	0.0000

*F*_3_	BPSO9	9.0000	2683.0000	1517.0000	890.0239
	BPSO10	0.0000	0.0000	**0.0000**	0.0000
	BPSO11	0.0000	0.0000	**0.0000**	0.0000
	BPSO12	0.0000	0.0000	**0.0000**	0.0000

*F*_4_	BPSO9	449.0000	2041.0000	1205.2000	434.1667
	BPSO10	449.0000	449.0000	**449.0000**	0.0000
	BPSO11	449.0000	449.0000	**449.0000**	0.0000
	BPSO12	449.0000	449.0000	**449.0000**	0.0000

*F*_5_	BPSO9	118.5000	124.5000	120.9000	1.9596
	BPSO10	112.5000	112.5000	**112.5000**	0.0000
	BPSO11	112.5000	112.5000	**112.5000**	0.0000
	BPSO12	112.5000	112.5000	**112.5000**	0.0000

*F*_6_	BPSO9	210.8445	1124.7927	618.0885	271.3089
	BPSO10	0.0001	0.0526	0.0216	0.0155
	BPSO11	0.0000	0.0132	0.0042	0.0039
	BPSO12	0.0004	0.0049	**0.0018**	0.0012

*F*_7_	BPSO9	1.0000	7.0000	4.9000	1.8682
	BPSO10	0.0000	0.0000	**0.0000**	0.0000
	BPSO11	0.0000	0.0000	**0.0000**	0.0000
	BPSO12	0.0000	0.0000	**0.0000**	0.0000

*F*_8_	BPSO9	8.8818*E* − 16	0.4927	0.3065	0.1329
	BPSO10	8.8818*E* − 16	8.8818*E* − 16	**8.8818*E* − 16**	0.0000
	BPSO11	8.8818*E* − 16	8.8818*E* − 16	**8.8818*E* − 16**	0.0000
	BPSO12	8.8818*E* − 16	8.8818*E* − 16	**8.8818*E* − 16**	0.0000

*F*_9_	BPSO9	1.2209	1.2811	1.2520	0.0176
	BPSO10	1.2108	1.2108	**1.2108**	0.0000
	BPSO11	1.2108	1.2108	**1.2108**	0.0000
	BPSO12	1.2108	1.2108	**1.2108**	0.0000

**Table 7:** Numerical statistics.

Function	Arithmetic	Best	Worst	Ave	Std
*F*_1_	BPSO1	20.0000	25.0000	23.7000	1.4177
	BPSO2	28.0000	34.0000	30.8000	1.8330
	BPSO3	36.0000	41.0000	38.4000	1.6852
	BPSO4	38.0000	44.0000	42.1000	1.5780
	BPSO5	2.0000	14.0000	7.7000	2.9343
	BPSO6	4.0000	9.0000	5.7000	1.5524
	BPSO7	3.0000	7.0000	5.0000	1.3416
	BPSO8	2.0000	7.0000	4.0000	1.4142
	BPSO10	0.0000	0.0000	**0.0000**	0.0000

*F*_2_	BPSO1	21.0000	28.0000	24.9000	1.9723
	BPSO2	28.0000	35.0000	32.5000	2.1095
	BPSO3	38.0000	42.0000	39.2000	1.1662
	BPSO4	38.0000	43.0000	41.4000	1.7436
	BPSO5	4.0000	12.0000	7.6000	2.3324
	BPSO6	4.0000	10.0000	6.8000	1.8868
	BPSO7	2.0000	7.0000	4.7000	1.6155
	BPSO8	1.0000	6.0000	3.8000	1.6000
	BPSO10	0.0000	0.0000	**0.0000**	0.0000

*F*_3_	BPSO1	2.0936*E* + 04	2.9895*E* + 04	2.4866*E* + 04	3.0074*E* + 03
	BPSO2	3.5023*E* + 04	4.7871*E* + 04	4.0582*E* + 04	4.5452*E* + 03
	BPSO3	4.0798*E* + 04	7.7036*E* + 04	6.3317*E* + 04	9.7782*E* + 03
	BPSO4	6.1630*E* + 04	8.7746*E* + 04	7.4415*E* + 04	7.9859*E* + 03
	BPSO5	9.2100*E* + 02	7.8520*E* + 03	3.2089*E* + 03	1.9484*E* + 03
	BPSO6	2.6700*E* + 02	4.6020*E* + 03	2.1907*E* + 03	1.3673*E* + 03
	BPSO7	9.2000*E* + 01	3.1230*E* + 03	1.0619*E* + 03	1.0267*E* + 03
	BPSO8	1.5600*E* + 02	2.5080*E* + 03	1.0006*E* + 03	8.4595*E* + 02
	BPSO10	0.0000	0.0000	**0.0000**	0.0000

*F*_4_	BPSO1	3651.0000	4256.0000	3899.0000	155.1722
	BPSO2	4072.0000	4657.0000	4406.2000	159.0540
	BPSO3	4163.0000	4971.0000	4750.7000	219.8095
	BPSO4	4771.0000	5162.0000	4963.3000	103.9173
	BPSO5	1828.0000	3047.0000	2207.5000	324.0522
	BPSO6	1937.0000	2454.0000	2135.4000	210.8327
	BPSO7	1643.0000	2650.0000	2179.6000	355.8585
	BPSO8	1640.0000	2848.0000	2175.5000	386.0337
	BPSO10	149.0000	149.0000	**149.0000**	0.0000

*F*_5_	BPSO1	85.5000	89.5000	87.5000	1.7889
	BPSO2	97.5000	107.5000	102.5000	3.6056
	BPSO3	105.5000	117.5000	112.3000	3.8158
	BPSO4	115.5000	125.5000	119.9000	3.2000
	BPSO5	37.5000	61.5000	52.1000	6.3906
	BPSO6	45.5000	53.5000	50.3000	2.4000
	BPSO7	41.5000	53.5000	47.7000	4.1425
	BPSO8	43.5000	47.5000	45.5000	1.7889
	BPSO10	37.5000	37.5000	**37.5000**	0.0000

*F*_6_	BPSO1	1174.2146	1680.9851	1489.1710	149.4409
	BPSO2	1760.8619	2317.8085	1949.8876	155.5235
	BPSO3	2496.6527	2707.5556	2584.3121	63.7418
	BPSO4	2691.9604	2984.0308	2850.7730	103.5472
	BPSO5	225.0052	567.0026	401.3030	109.7147
	BPSO6	162.0050	665.0021	346.3023	133.7193
	BPSO7	145.0009	504.0035	245.7022	113.2767
	BPSO8	144.0011	482.0005	297.3014	114.2555
	BPSO10	0.0001	0.0101	**0.0035**	0.0028

*F*_7_	BPSO1	22.0000	28.0000	25.3000	2.2383
	BPSO2	29.0000	34.0000	32.0000	1.4142
	BPSO3	36.0000	40.0000	38.1000	1.0440
	BPSO4	35.0000	44.0000	40.8000	2.7129
	BPSO5	4.0000	9.0000	6.4000	1.4283
	BPSO6	1.0000	11.0000	6.5000	2.6926
	BPSO7	2.0000	7.0000	4.8000	1.9391
	BPSO8	1.0000	6.0000	3.3000	1.4866
	BPSO10	0.0000	0.0000	**0.0000**	0.0000

*F*_8_	BPSO1	1.4420	1.5979	1.5153	0.0418
	BPSO2	1.6837	1.8667	1.7507	0.0513
	BPSO3	1.8418	1.9625	1.9171	0.0450
	BPSO4	1.9153	2.0532	1.9989	0.0400
	BPSO5	0.4566	1.0066	0.7834	0.1726
	BPSO6	0.6426	1.0066	0.8087	0.1022
	BPSO7	0.3239	0.8457	0.6732	0.1405
	BPSO8	0.3239	0.9028	0.6573	0.1738
	BPSO10	8.88178*E* − 16	8.88178*E* − 16	**8.8818*E* − 16**	0.0000

*F*_9_	BPSO1	1.9687	2.2083	2.0753	0.0814
	BPSO2	2.1886	2.4374	2.3050	0.0850
	BPSO3	2.5578	2.7476	2.6494	0.0629
	BPSO4	2.5683	2.7973	2.7120	0.0743
	BPSO5	1.3666	1.6074	1.4911	0.0771
	BPSO6	1.3666	1.5472	1.4309	0.0552
	BPSO7	1.3666	1.5773	1.4690	0.0634
	BPSO8	1.3365	1.4975	1.4068	0.0486
	BPSO10	1.2108	1.2108	1.2108	0.0000

*F*_10_	BPSO1	833.4458	897.9789	859.9714	23.8603
	BPSO2	797.1630	894.5194	855.8127	29.2840
	BPSO3	822.1992	935.9137	871.3066	31.0668
	BPSO4	801.6173	922.5607	860.1476	40.3582
	BPSO5	819.9967	922.3191	864.5066	32.5671
	BPSO6	798.7684	903.6756	848.3581	32.5885
	BPSO7	803.2907	904.1978	862.3321	35.3385
	BPSO8	833.7166	898.4921	865.7664	24.2585
	BPSO10	732.3788	790.8776	**764.5206**	20.0896

*F*_11_	BPSO1	811.5518	896.1991	860.3674	26.4694
	BPSO2	797.1630	905.6025	854.5382	29.9453
	BPSO3	822.1992	935.9137	862.4550	36.2997
	BPSO4	801.6173	888.0983	849.7746	28.6370
	BPSO5	819.9967	922.3191	868.3163	29.5430
	BPSO6	798.7684	903.6756	844.8258	35.6381
	BPSO7	803.2907	904.1978	865.2761	29.1488
	BPSO8	788.3447	898.6084	866.7004	37.5416
	BPSO10	732.3788	790.5087	**758.0837**	22.0786

*F*_12_	BPSO1	761.2894	912.1116	863.0355	43.0973
	BPSO2	803.8999	897.6278	850.4606	32.1798
	BPSO3	835.9801	912.0372	878.0874	23.8546
	BPSO4	772.1731	916.9646	858.7735	44.5248
	BPSO5	765.3802	900.9199	859.8082	41.3350
	BPSO6	810.4896	900.3629	856.8248	28.2321
	BPSO7	820.4377	918.6237	873.2900	30.3881
	BPSO8	811.3776	885.9333	852.6042	20.1360
	BPSO10	693.7516	811.1792	**758.9200**	38.4465

**Table 8:** Nonparametric test results.

	BPSO10vsBPSO1	BPSO10vsBPSO2	BPSO10vsBPSO3	BPSO10vsBPSO4	BPSO10vsBPSO5	BPSO10vsBPSO6	BPSO10vsBPSO7	BPSO10vsBPSO8
*F*_1_	5.721*E* − 05	6.386*E* − 05	6.069*E* − 05	4.880*E* − 05	5.893*E* − 05	6.069*E* − 05	5.849*E* − 05	5.763*E* − 05
*F*_2_	5.470*E* − 05	6.386*E* − 05	5.721*E* − 05	5.893*E* − 05	6.158*E* − 05	6.113*E* − 05	6.113*E* − 05	6.113*E* − 05
*F*_3_	6.386*E* − 05	6.386*E* − 05	6.386*E* − 05	6.386*E* − 05	6.386*E* − 05	6.386*E* − 05	6.386*E* − 05	6.386*E* − 05
*F*_4_	6.386*E* − 05	6.386*E* − 05	6.294*E* − 05	6.386*E* − 05	6.386*E* − 05	6.340*E* − 05	6.386*E* − 05	6.386*E* − 05
*F*_5_	5.470*E* − 05	5.849*E* − 05	5.721*E* − 05	6.113*E* − 05	2.238*E* − 04	5.980*E* − 05	6.203*E* − 05	5.470*E* − 05
*F*_6_	1.827*E* − 04	1.827*E* − 04	1.827*E* − 04	1.827*E* − 04	1.827*E* − 04	1.827*E* − 04	1.827*E* − 04	1.827*E* − 04
*F*_7_	6.113*E* − 05	5.849*E* − 05	5.470*E* − 05	6.113*E* − 05	6.113*E* − 05	6.249*E* − 05	6.069*E* − 05	5.849*E* − 05
*F*_8_	5.511*E* − 05	6.113*E* − 05	5.721*E* − 05	6.113*E* − 05	6.294*E* − 05	6.113*E* − 05	6.113*E* − 05	6.158*E* − 05
*F*_9_	6.340*E* − 05	6.294*E* − 05	6.340*E* − 05	6.294*E* − 05	6.249*E* − 05	6.158*E* − 05	6.294*E* − 05	5.893*E* − 05
*F*_10_	1.806*E* − 04	1.806*E* − 04	1.806*E* − 04	1.806*E* − 04	1.806*E* − 04	1.806*E* − 04	1.806*E* − 04	1.806*E* − 04
*F*_11_	1.827*E* − 04	1.827*E* − 04	1.827*E* − 04	1.827*E* − 04	1.827*E* − 04	1.827*E* − 04	1.827*E* − 04	2.461*E* − 04
*F*_12_	1.000*E* − 03	3.298*E* − 04	1.827*E* − 04	1.700*E* − 03	1.000*E* − 03	2.461*E* − 04	1.827*E* − 04	1.827*E* − 04

**Table 9:** Comparison results between BPSO10 and BPSOGSA.

Function		BPSO10	BPSOGSA [28]
*F*_1_	Ave	0.0000	0.7539
	Std	0.0000	0.7441

*F*_2_	Ave	0.0000	0.1584
	Std	0.0000	0.1219

*F*_3_	Ave	0.0000	45.2867
	Std	0.0000	94.4522

*F*_4_	Ave	74.0000	281.4150
	Std	0.0000	667.8743

*F*_5_	Ave	18.7500	8.0937
	Std	0.0000	17.6706

*F*_6_	Ave	0.0044	0.0064
	Std	0.0043	0.0089

*F*_7_	Ave	0.0000	1.8752
	Std	0.0000	1.2717

*F*_8_	Ave	8.8818*E* − 16	0.5412
	Std	0.0000	0.8005

*F*_9_	Ave	1.3744	0.3702
	Std	0.0000	0.4851

**Table 10:** Comparison results of BPSO10 with BHTPSO, BHTPSO-QI, and SOGA.

Function		BPSO10	BHTPSO [29]	BHTPSO-QI [29]	SOGA [30]
*F*_1_	Ave	0.0000	0.0010	0.0008	0.0002
	Std	0.0000	0.0029	0.0021	0.0005

*F*_2_	Ave	0.0000	0.0080	0.0082	0.0026
	Std	0.0000	0.0154	0.0153	0.0005

*F*_3_	Ave	0.0000	36.1500	35.0200	1919.6076
	Std	0.0000	66.3300	78.3500	1078.9650

*F*_5_	Ave	3.0000	0.0000	0.0000	0.1000
	Std	0.0000	0.0000	0.0000	0.4026

*F*_6_	Ave	0.0007	0.0028	0.0033	
	Std	0.0005	0.0020	0.0022	

*F*_8_	Ave	8.8818*E* − 16	−136.5000	−137.0000	1.3614
	Std	0.0000	1.7850	1.7240	1.1703

*F*_8_	Ave	2.4053			1.4639
	Std	0.0000			1.4386

## Data Availability

There are no data available for this paper.
